# H2-O deficiency promotes regulatory T cell differentiation and CD4 T cell hyperactivity

**DOI:** 10.3389/fimmu.2023.1304798

**Published:** 2024-01-05

**Authors:** Robin A. Welsh, Nianbin Song, Chan-Su Park, J. David Peske, Scheherazade Sadegh-Nasseri

**Affiliations:** Department of Pathology, Johns Hopkins School of Medicine, Baltimore, MD, United States

**Keywords:** immunology, class II antigen presentation, regulatory T cells, thymic selection, CD4 T cells

## Abstract

Regulatory T cells (Treg) are crucial immune modulators, yet the exact mechanism of thymic Treg development remains controversial. Here, we present the first direct evidence for H2-O, an MHC class II peptide editing molecular chaperon, on selection of thymic Tregs. We identified that lack of H2-O in the thymic medulla promotes thymic Treg development and leads to an increased peripheral Treg frequency. Single-cell RNA-sequencing (scRNA-seq) analysis of splenic CD4 T cells revealed not only an enrichment of effector-like Tregs, but also activated CD4 T cells in the absence of H2-O. Our data support two concepts; a) lack of H2-O expression in the thymic medulla creates an environment permissive to Treg development and, b) that loss of H2-O drives increased basal auto-stimulation of CD4 T cells. These findings can help in better understanding of predispositions to autoimmunity and design of therapeutics for treatment of autoimmune diseases.

## Introduction

T cells are key players in humoral immune responses. Upon infection with a pathogen, CD4 T cells utilize their T cell receptor (TCR) to survey for peptides bound to MHC class II molecules (pMHCII) presented by professional antigen presenting cells (APCs). Identification of cognate pMHCII complexes by the TCR leads to CD4 T cell activation and ultimately clearance of the foreign pathogen. Faulty activation, however, can lead to deleterious inflammation causing possible autoimmune diseases and cancer development. Hence, multiple regulatory processes exist to ensure T cell activation remains in check.

Regulation of T cell activation begins during thymic development where immature thymocytes are screened for self-reactivity. Broadly divided into positive and negative selection, this process ensures that CD4 T cells expressing high avidity self-reactive TCRs are either eliminated (1), or become CD4 regulatory T cells (Tregs) (2). MHC Class II antigen processing machinery expressed by medullary thymic epithelium cells (mTECs) and thymic antigen presenting cells (APCs) are critical for screening for auto-reactive T cells. Two chaperone proteins, H2-M (murine; human, HLA-DM), and H2-O (murine; human, HLA-DO) are major components of the MHC II processing pathway. While H2-M is expressed in all APCs, H2-O is expressed in the thymic medulla, B cells and various dendritic cell subpopulations (3, 4). H2-M plays a critical role in MHC class II antigen processing by dissociating the Class II Invariant Chain peptide (CLIP) from the newly synthesized MHC II. Dissociation of CLIP promotes a peptide-receptive MHC II conformation to which denatured protein antigens can be screened for the best MHC II groove fitting epitopes. However, a peptide-receptive MHC II conformation is highly transient and in the absence of suitable peptides readily reverts to a closed conformation (5–8). We have proposed that H2-O binds to MHC II in receptive conformation (9) and works cooperatively with H2-M to stabilize the peptide-receptive MHC II conformation for an optimized epitope selection process (10). Together, H2-M and H2-O molecules can ensure that the best MHC II groove fitting epitopes are selected for presentation to cognate CD4 T cells.

While the exact mechanism of Treg selection remains to be fully understood (11), two critical requirements have been recognized as necessary for a successful thymic Treg development. First, thymic Treg development requires relatively strong TCR signaling in the thymic medulla (12), and second, Treg development relies on signaling by the common γ chain (γC) cytokines, mainly IL-2, for driving Foxp3 expression (13). While a strong TCR signaling during negative selection normally leads to CD4 T cell deletion (1), a somewhat weaker TCR signaling has been suggested to promote Treg selection (11, 14, 15). TCR signaling is affected by both the nature and density of the presented self-peptides (16, 17). If epitopes are in high abundance and more ubiquitously expressed in the medulla, then cognate CD4 T cells will undergo clonal deletion. However, if epitopes are in lower abundance and have a sparser expression, leading to discontinuous TCR stimulation, then cognate CD4 T cells might undergo Treg selection. This model of Treg selection relies on the level of TCR signaling that medulla localized CD4 single-positive (SP) T cells receive. As loss of H2-O in naïve peripheral B cells has been shown to alter both the repertoire and density of presented peptides (18, 19) we questioned if loss of H2-O in the thymus could affect whether CD4 T cells are signaled for deletion, or survival during thymic deletion. Should a lower density of self pMHCII be presented in the thymic medulla, an increased number of self-reactive CD4 T cells might escape deletion leading to an increased frequency of auto-reactive T cell clones in the periphery. Or, alternatively, presentation of a lower density of self pMHCII could promote selection of regulatory T cells. Here, we demonstrate that loss of H2-O generates a more stimulatory *in vivo* environment impacting both the thymic development and peripheral activation of regulatory T cells.

## Results

### Loss of H2-O increases the activation state of auditing medulla CD4 T cells

**Figure 1:**
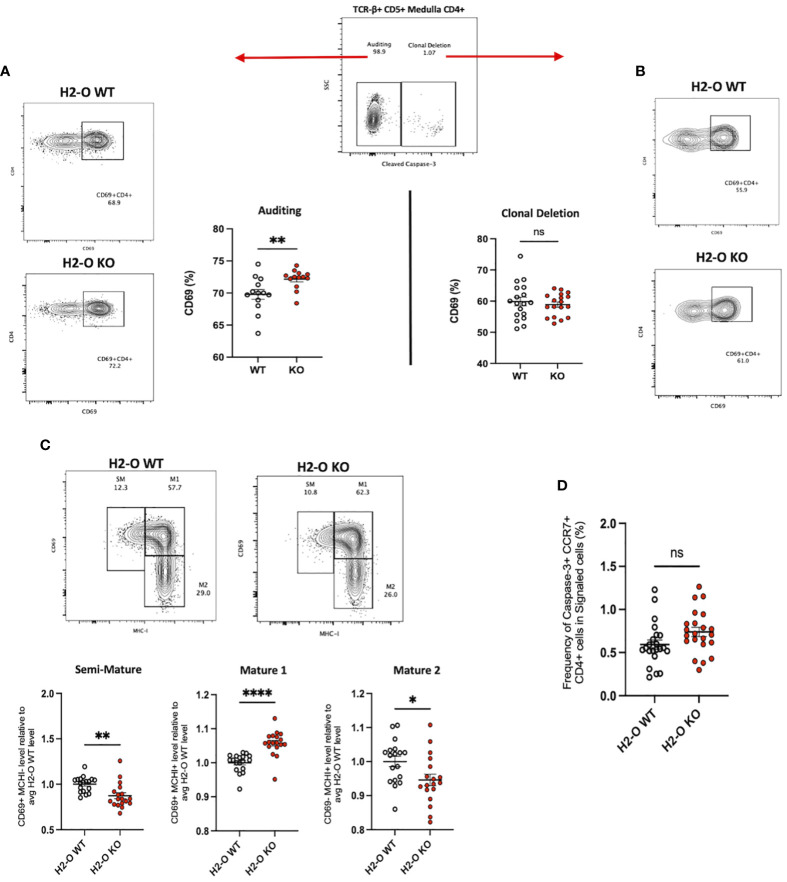
Loss of thymic H2-O increases the activation state of auditing CD4 T cells **(A)** Left: representative contour plots showing total CD69 expression in auditing (Caspase-3 negative) signaled (TCR-B+CD5+) CCR7+CD4+ T cells from 6-week-old H2-O WT (Top) and H2-O KO (Bottom) thymi. Right: Combined CD69 expression data from 5 repeat experiments. N= 18 mice per genotype **(B)** Right: representative contour plots showing total CD69 expression in Clonally deleted (Caspase-3 positive) signaled (TCR-B+CD5+) CCR7+CD4+ T cells from 6-week-old H2-O WT (Top) and H2-O KO (Bottom) thymi. Left: Combined CD69 expression data from 5 repeat experiments. N= 18 mice per genotype **(C)** Top: representative contour plots showing the subdivision of auditing (Caspase-3 negative) signaled (TCR-B+CD5+) CCR7+CD4+ T cells from 6-week-old H2-O WT (Left) and H2-O KO (Right) thymi into three maturation stages: Semi-Mature (SM), Mature 1 (M1), and Mature 2 (M2). Bottom: Cumulative maturation state data from 5 repeat experiments, N = 18 mice per genotype. Expression has been normalized to the average H2-O WT levels within each experiment to allow for comparison across experiments. Raw percentage data can be found in **Supplementary Figure 3**. **(D)** Frequency of medulla specific (CCR7+) CD4 T cells selected for clonal deleted (Caspase-3+) ns, not significant,*<0.05, **<0.001, ***<0.0001, ****<0.00001 Statistics: unpaired student T-test.

Previously, we demonstrated that loss of H2-O expression correlated with both an increased B cell presentation of low-affinity MHC II peptides, and an increased frequency of a MOG_35-55_ specific, self-reactive CD4 T cell (18). Because of H2-O expression in the medullary thymus we speculated that H2-O deficiency might lead to presentation of lower densities of high-affinity self-peptides in the medulla, thereby causing altered clonal deletion. Based upon findings by Breed et al. positively selected (TCR-β+ CD5+) medulla CD4 T cells can be subdivided into two main populations, “Auditing” (CCR7+ Caspase-3_neg_) and “Clonally Deleted” (CCR7+ Caspase-3_pos_) T cells (20). Using this strategy, we examined the levels of positively selected (TCR-β+CD5+) CD4 T cells undergoing auditing (CCR7+ CD4+Caspase-3_neg_) or clonal deletion (CCR7+ CD4+Caspase-3_pos_) in 6 week old male and female H2-O WT and H2-O mice (**Figure 1**; **Supplementary Figure 1**). Loss of H2-O was found to significantly increase the expression of the activation marker CD69 on CD4 T cells undergoing active self-auditing (**Figure 1A**), but not those selected for clonal deletion (**Figure 1B**).

CD69 in combination with MHC-I defines 3 medullary maturation stages: semi-mature (CD69+ MHC-I -), mature 1 (CD69+ MHC-I +), and mature 2 (CD69- MHC-I -) (20). Subdivision of auditing H2-O-KO CD4 T cells identified a significant increase in CD4 T cells with a Mature 1 phenotype (**Figure 1C** middle). Conversely, both the semi-mature and mature 2 stages were decreased in H2-O KO mice (**Figure 1C** left/right). No differences in any maturation stage were found in the clonally deleted CD4 T cell population (**Supplementary Figure 2** **).** Furthermore, H2-O deficiency, did not appear to alter the rate of CD4 T cells undergoing clonal deletion (**Figure 1C**). Importantly, no differences were observed in thymocytes undergoing positive selection (**Supplementary Figure 3**). These data suggest that loss of H2-O drives a more stimulatory thymic medulla environment, but with similar levels of clonal deletion. It is therefore likely that the increased peripheral frequencies of MOG-specific CD4 T cells previously identified is due to increased peripheral expansion of the MOG-reactive clone, not a general alteration in clonal deletion.

### H2-O KO thymi have increased regulatory T cell development

**Figure 2:**
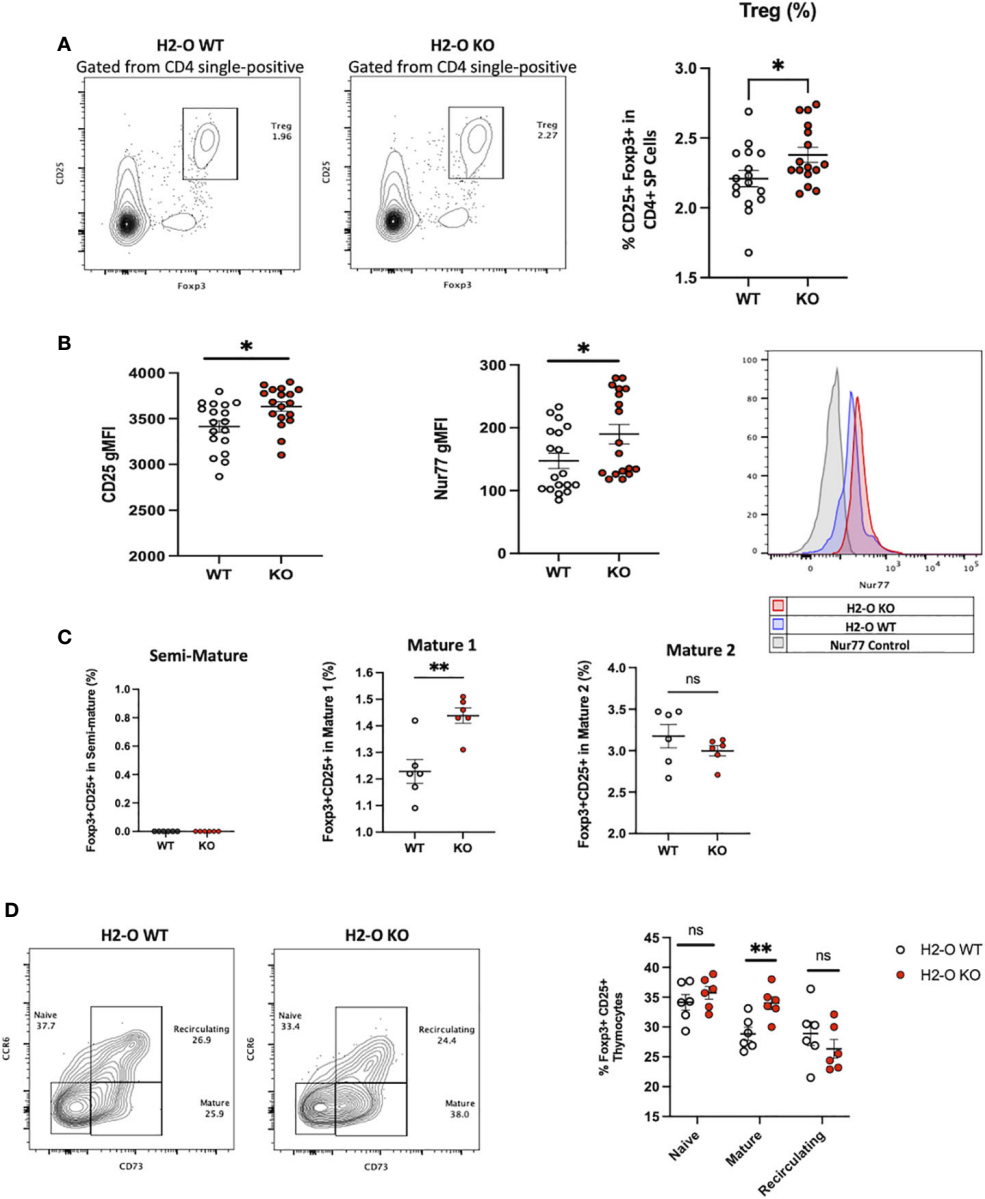
H2-O KO thymi have increased regulatory T cell levels. **(A)** Left: representative contour plots showing the frequency of Tregs (CD25+ Foxp3+) cells in the CD4 single-positive thymus population. Right: cumulative percentage of Foxp3+ CD25+ cells within the CD4 single-positive thymus population in H2-O WT (white) or H2-O KO (red) cells. Data from 3 replicate experiments, N= 16 mice per genotype. **(B)** Geometric mean fluorescence intensity (gMFI) of CD25 (left), Nur77 (right) expressed by Foxp3+ CD4+ T cells in the thymus of H2-O WT (white) or H2-O KO (red) mice. **(C)** Subdivision of Treg cells into three maturation stages: Semi-Mature (Left), Mature 1 (Middle), and Mature 2 (Right). Data from 2 replicate experiments. **(D)** Left: representative contour plots showing the frequency of Naive (CCR6- CD73-), Mature (CCR6- CD73+), and Recirculating (CCR6+ CD73+) Foxp3+ CD25+ Tregs in 6-week-old H2-O WT and H2-O KO thymi. Right: Summary plots showing the frequency of Naive (CCR6- CD73-), Mature (CCR6- CD73+), and Recirculating (CCR6+ CD73+) Foxp3+ CD25+ Tregs from 2 independent repeat experiments, N= 6 mice per group. ns = not significant, * = <0.05, ** = <0.001. Statistics: unpaired student T-test.

With increased levels of peripheral Tregs previously identified in H2-O KO mice (18), we also questioned if H2-O deficiency was affecting Treg selection. In fact, one model of thymic Treg selection centers around the concept of antigen density (14, 21). Within this “mosaic” model, sporadic MHC-TCR interactions with sparsely presented self-epitopes leads to Treg development. Since it has been shown that peripheral loss of H2-O leads to alterations in peptide presentation (18, 19, 22), we postulated that altering the level of self-epitopes present in the medulla could alter Treg selection. Analysis of CD4 single-positive T cells identified an increased frequency of CD25+Foxp3+ T cells in H2-O KO mice (**Figure 2A**). Furthermore, H2-O KO thymic Tregs (tTregs) expressed higher levels of the high affinity IL-2 receptor (CD25) (**Figure 2B**/left), and the orphan nuclear receptor Nur77 (**Figure 2B**/right). As Nur77 has been associated with the level of TCR engagement (23), increased Nur77 expression strongly indicates that absence of H2-O leads to increased TCR engagement suggesting an increased self-reactivity. Finally, maturation state analysis found that H2-O KO tTregs were enriched in the M1 stage (**Figure 2C**).

Since peripheral Tregs are known to recirculate back to the thymus (24) we investigated what percentage of the identified tTreg pool in H2-O WT and H2-O KO thymi came from the periphery. As show in **Figure 2D**, similar levels of recirculating (CCR6+ CD73+) Tregs were identified in both H2-O WT and H2-O KO thymi. Supporting the identification of increased M1 stage Tregs, CCR6- CD73+ (mature) Tregs were statistically increased in H2-O KO mice. No major differences were found in the level of naïve (CCR6- CD73-) Tregs. These findings suggest that the increased Treg levels observed in H2-O KO thymi are likely due to an increased *de novo* Treg selection not an increased recirculation of peripheral Treg.

### Loss of H2-O correlates with increased peripheral CD4 T cell activation

**Figure 3:**
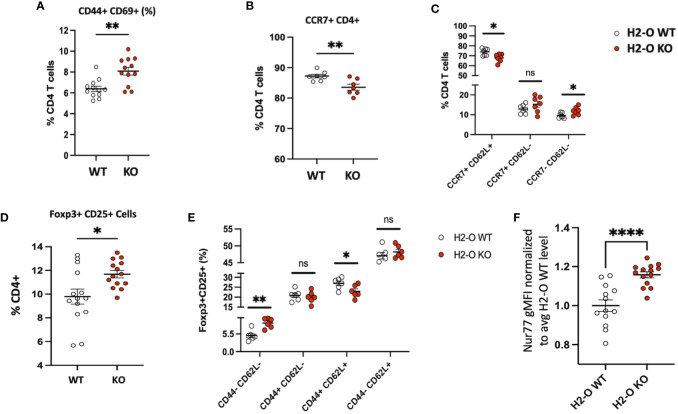
H2-O KO mice have an increased activated peripheral CD4 T cell population **(A)** Basal levels of CD44 and CD69 expressed by H2-O WT (white) and H2-O KO (red) splenic CD4 T cells. Increased CD69 expression, a marker of recent activation showed increased levels on H2-O KO CD4 T cells **(B)** Percentage of CD4+ T cells expressing the lymphoid tissue homing receptor CCR7 **(C)** CCR7 and CD62L expression levels in unimmunized splenic CD4 T cells **(D)** Frequency of Foxp3+CD25+ cells in splenic CD4+ T cell population of unimmunized mice **(E)** H2-O KO peripheral Tregs express decreased levels of naïve (CD44+ CD62L+) expressing cells **(F)** Normalized Nur77 gMFI levels in Foxp3+CD25+ Treg cells in H2-O WT (white) and H2-O KO (red) cells. To account for experimental variation the average Nur77 gMFI level in H2-O WT samples was calculated. gMFI levels in both H2-O WT and H2-O KO samples were then divided by the calculated H2-O WT average. An increased Nur77 ratio indicates increased Nur77 gMFI levels. Summary of 3 repeat experiments. *<0.05, **<0.001, ***<0.0001,****<0.00001.

Considering the observation that lack of H2-O did not appear to alter clonal deletion frequencies but did affect the level of thymic CD4 T cell activation, we next evaluated whether peripheral loss of H2-O also increased CD4 T cells activation. Unimmunized H2-O KO spleens had an increased frequency of CD4 T cells co-expressing the key activation markers CD44+ and CD69+ (**Figure 3A**) and the tissue homing marker CCR7 (**Figure 3B**). We further assessed the levels of “non-activated” (CCR7+ CD62L+) versus “activated” (CCR7- CD62L-) CD4 T cells (25), and found lower frequencies of non-activated CD4 T cells in H2-O KO mice (**Figure 3C**, left). Importantly, this correlated with an increase in percentage of activated CD4 T cells (**Figure 3C**, right). Collectively, these phenotypic analyses support the idea that loss of H2-O leads to increased basal levels of activated CD4 T cells in unimmunized H2-O KO mice.

As discussed above, H2-O KO thymi promoted Tregs selection. Consistent with these observations and our previously published data (18, 26), H2-O KO spleens had an increased frequency of CD25+ Foxp3+ Tregs (**Figure 3D**). Furthermore, H2-O KO Tregs showed decreased levels of CD62L (**Figure 3E**) and increased levels of Nur77 (**Figure 3F**) indicating a larger proportion of Tregs cells are likely more activated and circulating through the periphery of H2-O KO mice.

### Single cell RNA-sequencing of H2-O KO splenic CD4 T cells confirms increased activation

**Figure 4:**
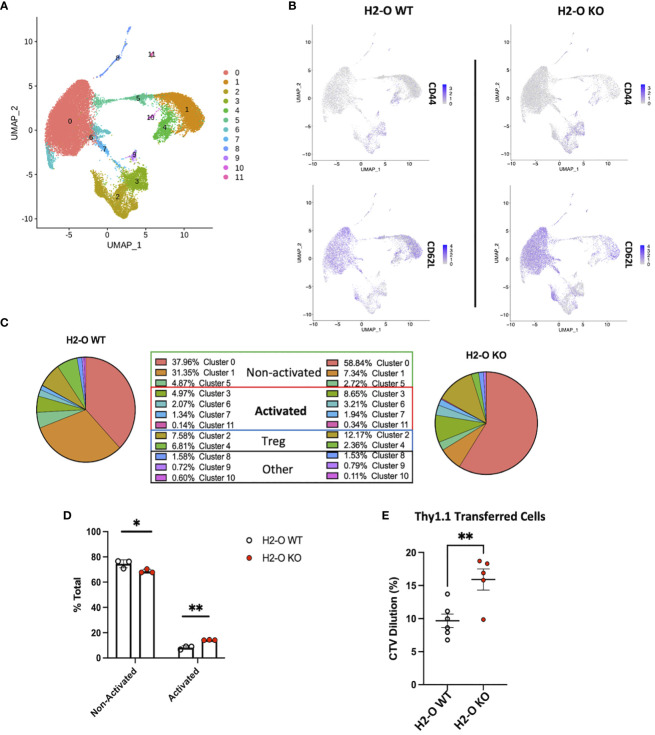
Loss of H2-O function causes increased basal CD4 cell activation. **(A)** scRNA-seq clustering of CD4 T cells after Seurat analysis. Data represents the average of of 3 biological replicates per genotype. **(B)** Co-expression of CD44 (top) and CD62L (bottom) within either H2-O WT (left) or H2-O KO (right) clusters. **(C)** Breakdown of clusters in H2-O WT (Left) or H2-O KO (Right) samples. Clusters are grouped based upon, (1) known CD4 T cell subset markers and (2) gene comparison to published CD4 T cell data sets available on the Immunological Genome Project (www.immgen.org). Identified CD4 Cell phenotypes were: Non-activated, Activated, and Regulatory T cells. ”Other” refers to a minor macrophage and NKT cell contamination from the sorting process. **(D)** Distribution of the for the Non-activated and Activated phenotypes across the H2-O WT and H2-O KO biological replicates (N = 3 mice per genotype). **(E)** *In vivo* proliferation of adoptively transferred naïve Thy1.1+ CD4 T cells (Thy1.1+ CD3+ CD4+ CD44- CD25-) after 7 days in either Thy1.2 H2-O WT (white) or Thy1.2 H2-O KO (red) hosts. Pooled data from 2 independent experiments (N= 6 H2-O WT, 5 H2-O KO). ns = not significant, * = <0.05, ** = <0.001, ***= <0.0001, **** = <0.00001. Statistics: unpaired student T-test.

Based upon the strong FACS data above exhibiting increased numbers of Tregs, and more activated CD4 T cells, we attempted single-cell RNA-sequencing (scRNA-seq) to gain a more holistic unbiased characterization. CD3+ CD4+ NK1.1- CD19- cells were sorted from spleens of 3 unimmunized H2-O WT and 3 unimmunized H2-O KO mice and subjected to 10x Genomics scRNA-seq analyses. In total, 11 distinct CD4 T cell clusters were identified (**Figure 4A**; **Supplementary Figure 4**). Separation of the clusters based upon H2-O expression identified a dramatic increase in cluster 0, cluster 2 and cluster 3 in the H2-O KO samples. Conversely, clusters 1 and 4 were significantly increased in H2-O WT samples. Further refinement based upon expression of CD44 and CD62L (Sell) showed that most of clusters 0, 5 and 1 were naïve (CD44- CD62L+) CD4 T cells and CD44+CD62L- effector CD4 T cells were mainly located in clusters 2, 3, and 4. **Supplementary Figure 5** shows key genes significantly upregulated in Clusters 0, 1, 2, 3, and 4. No detectable difference was observed in cells expressing central memory markers (CD44+ CD62L+) within Cluster 0 (**Figure 4B**).

Comparing the genes from Clusters 0, 1 and 5 to the published CD4 T cell datasets using the “My Geneset” function on the Immunological Genome Project (www.immgen.org) suggested that upregulated genes (Log_2_Avg Fold-change (FC) 0.5) were present in both “naïve” and “activated” CD4 T cell datasets (**Figure 4C**). Based upon the high expression of CCR7 however, these clusters were labeled as “non-activated”. Clusters 3, 6, 7, and 11 aligned with mainly an “activated” CD4 T phenotype. Cluster 3 was found to have increased expression of the activation marker CD44, while Cluster 11 expressed high Ki67. Clusters 2 and 4 contained the known Treg genes Foxp3 and Il2ra (CD25) (**Supplementary Table 1**). Clusters 8, 9, and 10 were small populations of Macrophages and NKT cell (**Supplementary Table 1**). Condensing the cluster analysis further supported our initial FACS data that unimmunized H2-O-KO mice have a decreased frequency of “Non-activated” CD4 T cells (68.90% avg H2-O KO vs 74.17% avg H2-O WT with a coinciding increase in “Activated” (14.14% avg H2-O KO vs 8.53% avg H2-O WT) CD4 T cells (**Figure 4D**).

To test whether the higher activated state in H2-O KO T cells were possibly induced by the *in vivo* environment, we performed an adoptive transfer experiment of Treg depleted naïve Thy1.1+ CD4+ T cells from H2-O WT mice into either unimmunized Thy1.2+ H2-O WT, or Thy1.2+ H2-O KO hosts. Seven days post-transfer we observed that H2-O KO hosts induced significantly more *in vivo* proliferation of the Thy1.1+ WT donor cells as compared to the H2-O WT hosts (**Figure 4E**). These data further support our working model that loss of H2-O promotes increased T cell activation by APCs presenting a wider range of self-epitopes (18).

### H2-O KO Tregs have a more activated phenotype

**Figure 5:**
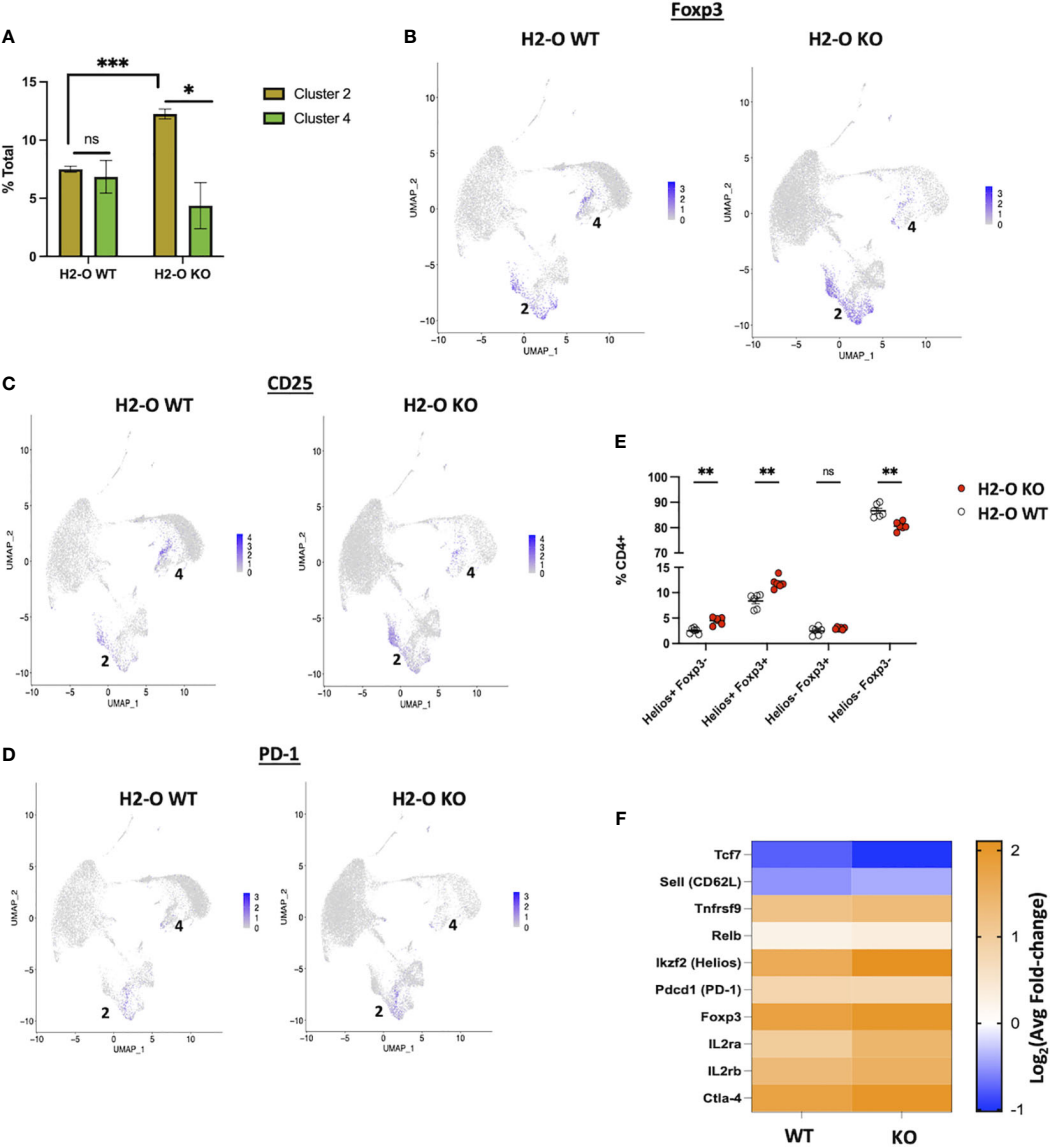
The H2-O KO Treg population has a more effector like phenotype. **(A)** (Left) distribution of the two scRNA-seq Treg clusters in either H2-O WT (left) or H2-O KO (right) samples. (Right) Expression of key Treg phenotypic markers: Foxp3 (Top) and CD25 [Il2ra] (Bottom). **(B)** Expression of Foxp3 in H2-O WT (Left) or H2-O KO (Right) clusters. **(C)** Expression of CD25 (IL2ra) in H2-O WT (Left) or H2-O KO (Right) clusters. **(D)** Expression of PD-1 (Pdcd1) in H2-O WT (Left) or H2-O KO (Right) clusters. **(E)** Subdivision of the splenic CD4+ T cell population by Foxp3 and Helios expression. **(F)** Comparison of cluster 2 upregulated genes (Log2Avg FC >0.5) in H2-O WT and H2-KO samples to a published (Miragaia et al.) splenic effector Treg genetic profile. ns = not significant, * = <0.05, ** = <0.001, ***= <0.0001. Statistics: unpaired student T-test.

Initial analyses of the two Treg clusters revealed that the frequency of Clusters 2 and 4 were roughly equal in H2-O WT samples, whereas H2-O KO samples had an overrepresentation of Cluster 2 (**Figure 5A**). Further analysis showed that both clusters 2 and 4 expressed the classic Treg genes, Foxp3 (**Figure 5B**), IL2ra [CD25] (**Figure 5C**), Pdcd1 [PD-1] (**Figure 5D**), and CTLA-4 (**Supplementary Table 1**). However, we noted that the Foxp3+ cells in cluster 2 segregated into two populations based upon CD25 expression, Foxp3+ CD25^Low^ and Foxp3+ CD25+, with a distinct enrichment of the Foxp3+ CD25^Low^ populations in H2-O KO samples. This was noteworthy as it has been shown that CD25-low FOXP3+ Treg are associated with a more inflammatory state (27). Cluster 2 analysis also identified a small number of cells that expressed the Th17 transcription factor Rorc (RORy), but only in cells from H2-O KO mice (**Supplementary Figures 5, 6**). These findings support the association of H2-O with a less inflammatory environment.

Also identified with the Treg clusters was the transcription factor Ikzf2 [Helios], a marker of stable Treg lineage commitment in inflammatory conditions (28), and a controversial marker of thymic Tregs (29–32). FACS analysis of Helios protein expression not only supported the scRNA-Seq data that both H2-O WT and H2-O KO CD4 Tregs express Helios but also revealed that H2-O KO mice have an increased frequency of Helios+Foxp3+ Tregs (**Figure 5E**). Since UMAPs preserve information about the distance between clusters (33) we hypothesized that Tregs from Cluster 2 were transcriptional distinct from those in Cluster 4. Comparison of the Cluster 2 genetic signature with a published Treg data set (34) indicated that these cells are likely effector-like Tregs. Furthermore, breaking down Cluster 2 showed that H2-O KO Tregs had a larger fold change in expression of the core effector gene signature (**Figure 5F**). We propose that this activated Treg state is likely driven by the increased basal CD4 T cell activation. Expansion of this Treg population could certainly be a mechanism controlling spontaneous autoimmunity in H2-O KO mice.

In summary, both scRNA-seq and FACs analyses showed that H2-O KO mice have an increased frequency of both conventional and regulatory CD4 T cells in the spleens of unimmunized mice. Loss of thymic H2-O expression correlated with an increased tTreg population that was not from peripheral recirculation and have received increased TCR stimulation which suggests increased autoreactivity. Furthermore, increased activation of conventional splenic CD4 T cells is likely a driving factor for the increased effector-like Treg status that was identified by scRNA-seq analyses of H2-O-KO CD4 cells.

## Discussion

In this study, we have refined our understanding of how loss of H2-O alters CD4 T cell activity *in vivo*. Thymic analysis of medulla localized CD4 T cells pointed to a novel role for H2-O on CD4 T cells undergoing self-auditing. Importantly, lack of H2-O expression led to an increased frequency of both thymic and peripheral Tregs.

Tregs play a fundamental role in maintenance of homeostasis, hence a better understanding of the mechanism of their development is highly desirable and the subject of numerous studies (14, 21). Our studies here are the first attempt in demonstrating that H2-O is a critical player in Treg development. While the exact mechanism by which thymic Treg cells are selected remains somewhat uncertain, recent studies support the idea that intermittent TCR signaling along with cytokine signaling drive Treg development (15). These findings and others (35–39) support a more avidity based model of Treg selection, in which alterations to the density of self-ligands present in the medulla will have differential effects on CD4 T cell selection. Intermittent TCR signaling as the driving force in Treg selection is very relevant to our understanding of how HLA-DO contributes to epitope selection during antigen processing. HLA-DO in complex with HLA-DM leads to better refinement of the epitopes from denatured antigens, promoting selection of the best fitting epitopes in the groove of MHC II molecules (10, 40). While this idea conflicts with the original model of how DO functions (41), recent studies using multiple human HLA-DO variants showed that certain variants enhanced DM activity (42). As such, when DO is present, higher affinity peptides are more likely to be presented. Conversely, in the absence of DO a larger percent of lower affinity epitopes are selected. Indeed, we have recently reported that peptides eluted from H2-O KO mice expressing either murine I-A^b^ (18) or human HLA-DR1 (Welsh et al, unpublished data) were of a lower general affinity. Accordingly, presentation of a larger portion of lower affinity peptides in the thymic medulla of H2-O KO mice is possible. Intermittent TCR signaling is typically generated by either pMHCII that are less stable (16, 43–45), or TCR/pMHC of lower affinity (46). Our findings here support the former. We associate increased Treg development as a consequence of the presentation of lower affinity peptides to self-auditing CD4 T cells.

Due to the continuous egress of mature CD4 T cells from the thymus, we also analyzed splenic CD4 T cells from unimmunized H2-O WT and H2-O KO mice. H2-O KO splenic CD4 T cells in unimmunized mice revealed an increased frequency of CD44hiCD69+ antigen experienced CD4 T cells, which suggests an increased level of antigen-specific TCR signaling in H2-O KO mice. As previously implied (47), a simple explanation for this activated state is incomplete thymic deletion in H2-O KO mice. While we were able to show that H2-O KO mice failed to delete specific CD4 clonal populations (18), detection of differences at the global levels did not show significant changes between the two genotypes. Nonetheless, we suggest that presentations of different arrays of self-antigens as well as their lower densities on thymic medulla leads to a less than optimal thymic deletion of self-reactive CD4 T cells and their routing to the periphery. Similar to activated CD4 T cells in the periphery, H2-O KO Treg cells also had a more effector-like phenotype, indicating enhanced Treg activation in unimmunized mice (34, 48).

In conclusion, our studies add a new dimension to our understanding of the role of H2-O in both CD4 T cell selection and activation. For the first time, we report on thymic negative selection in H2-O KO mice and demonstrate that loss of H2-O enhances thymic selection of regulatory T cells. Once in the periphery, an increased proportion of H2-O KO Tregs appear to be activated in a TCR dependent manner. These effector-like Tregs will likely help control increased basal CD4 T cell activation. However, the exact mechanism by which increased Treg cells are selected in the H2-O KO thymus remains to be determined. While we propose that alterations in medulla pMHCII-TCR avidity interactions could lead to enhanced Treg selection, increased presentation of self-antigens in a more tissue restricted antigenic (TRA) manner could also be possible (14).

## Methods

### Mice

Male and female C57BL/6J (H2-O WT, stock no: 000664), Female B6.PL-Thy1a/CyJ (stock no: 000406) were purchased from Jackson Laboratories and bred in house. Generation of the H2-O knock-out mice has been previously described (49) and mice bred in house. Unless otherwise stated analyzed mice were 6-8 weeks. All mouse procedures were approved by the Johns Hopkins University Animal Care and Use Committee and were following relevant ethical regulations.

### Antibodies/reagents

Flow Cytometry: anti-CD3e (17A2), anti-CD4 (RM4-5), anti-CD5 (53-7.3), anti-CD8α (53-6.7), CD11c (N418), anti-CD19 (ID3), anti-CD25 (PC61), anti-CD44 (IM7), anti-CD45R/B220 (RA3-6B2), anti-CD62L (MEL-14), anti-CD69 (H1.2F3), anti-CD197/CCR7 (4B12), anti-NK1.1 (PK136), anti-TCRβ (H57-597), anti-TCRγδ (GL3), anti-Cleaved Caspase 3 (D3E9) Cell signaling (Danvers, MA); Foxp3 (150D), anti-Helios (22F6), anti-Nur77 (12.14), Fixable viability dye eFluor 780 (eBioscience).

FACs Sorting: anti-CD3e, anti-CD4, anti-CD19, anti-NK1.1, Propidium iodide. Briefly, 30,000 live CD4 T cells [Gating: CD3+, CD4, CD19-, NK1.1-, PI-] were FACs sorted from the spleens of 3 unimmunized 10 week-old H2-O WT and 3 unimmunized 10 week-old unimmunized H2-O KO female mice.

### Cell staining

For cleaved caspase 3 staining (20) homogenized mice thymocyte cells were stained with anti-CCR7/CD197 at a final dilution of 1:50 for 30 min at 37°C prior to additional surface stains. Following surface staining, cells were fixed with Cytofix/Cytoperm (BD Biosciences) for 20 min at 4°C. Cells were then washed with Perm/Wash buffer (BD Biosciences) twice. Cells were stained with anti–cleaved caspase 3 at a 1:50 dilution at 23°C for 30 min.

For transcription factor staining, cells were incubated with surface antibody at 4°C for 20 min, permeabilized at 4°C for 30 min using a Foxp3/Transcription factor buffer set (Invitrogen, ThermoFisher Scientific), and then stained with anti–Foxp3 and/or anti-Helios at 23°C for 30 min.

### Adoptive cell transfer

Naïve CD4 T cells were isolated from the pooled spleens and LNs of 4 week old Thy1.1 expressing C57BL/6J mice using the EasySep Mouse Naive CD4 T cell Isolation Kit (StemCell Technologies). Isolated cells were stained with eFluor 450 viability dye (eBiosciences) according to manufactures directions. 3x10^6^ dye labeled Thy1.1 cells were IV injected into 10 week old H2-O WT or H2-O KO hosts. Transferred cells were recovered 7 days post-transfer.

### scRNA-sequencing

#### Library & sequencing

The samples were prepared using the 10x Genomics Chromium Next GEM Single Cell 5’ Library and Gel Bead Kit v1.1, Chromium Next GEM Chip and Dual Index Kit TN Set A. They were run on the Illumina NovaSeq6000 with a run configuration of 28bp x 10bp x 10bp x 91bp.

#### Analysis

Analyses of T-cell scRNA-seq were performed with the package Seurat (50), as follows. Data was filtered to remove cells with low gene count (<200), large number of UMIs (>12,000) and high (>5%) fraction of mitochondrial reads. Expression levels of genes were log-normalized, and the most variable 2000 genes were selected for linear dimensionality reduction with Principal Component Analysis (PCA). The first 15 principal components were then used to performed unsupervised clustering using the Seurat SNN clustering package, with a resolution of 0.2. To identify cell types, potential markers for each cluster were calculated as the set of genes significantly differentially expressed in each cluster compared to all others, using the function FindAllMarkers, and by searching existing literature and marker databases. Lastly, differentially expressed genes for each cell type between conditions were determined using the function FindMarkers with the default function (bimod).

Defining clusters – Genes with an average fold-change (avgFC) >0.5 and an adjusted p-value <0.05 were run against CD4 T cell data sets available on the Immunological Genome Project (https://www.immgen.org/) using the “My Geneset” data browser function.

#### Statistical testing

GraphPad Prism was used for all statistical analyses. A standard Student T-test was used for estimation of statistical significance. Data is shown as mean ± SEM. *p<0.05, **p<0.01, ***p<0.001, ****p<0.0001.

## Data availability statement

The scRNA-Seq data presented in the study are deposited in the NCBI GEO repository, accessionnumber: GSE246253.

## Ethics statement

The animal study was approved by Johns Hopkins Animal Care and Use Committee. The study was conducted in accordance with the local legislation and institutional requirements.

## Author contributions

RW: Conceptualization, Data curation, Formal analysis, Investigation, Methodology, Project administration, Resources, Validation, Visualization, Writing – original draft, Writing – review & editing. NS: Investigation, Methodology, Resources, Validation, Visualization, Writing – review & editing. C-SP: Conceptualization, Methodology, Visualization, Writing – review & editing. JP: Conceptualization, Investigation, Writing – review & editing. SS-N: Conceptualization, Data curation, Funding acquisition, Methodology, Project administration, Resources, Supervision, Visualization, Writing – original draft, Writing – review & editing.

## Supplementary material

Supplementary Figure 1 Gating strategy used for detection of Auditing *vs* Clonally Deleted Medulla CD4 T cells Red arrows indicate how Auditing *vs* Clonal Deletion was identified in 6-week-old H2-O WT and H2-O KO thymi. Blue arrows indicate additional gating corresponding to . Black Arrows indicate additional gating corresponding to (**Figure 1B**).

Supplementary Figure 2 The frequency of auditing Mature 1 CD4 T cells was increased in H2-O KO thyme **(A)**. Percentage of auditing (Caspase-3 negative) CD4 T cells in the Semi-mature (CD69+ MHC-I neg), Mature 1 (CD69+ MHC-1+), and Mature 2 (CD69- MHC-I+) in H2-O WT (white) and H2-O KO (red) mice N= 18 mice per group **(B)**. Percentage of clonally deleted (Caspase-3+) CD4 T cells in the Semi-mature (CD69+ MHC-I neg), Mature 1 (CD69+ MHC-1+), and Mature 2 (CD69- MHC-I+) in H2-O WT (white) and H2-O KO (red) mice. N= 18 mice per group Statistical Testing: unpaired student T-test.

Supplementary Figure 3 Positive Selection is not affected by loss of H2-O **(A)**. Representative plots showing CD4, CD8 and Double Positive (DP) percentages in H2-O WT (Top) and H2-O KO (Bottom) **(B)**. Summary plots of the single-positive CD4 (Top) and CD8 (Bottom) percentages from 5 repeat experiments. **(C)**. (Left) representative flow plots showing the “Signaled” *vs* “Non-signaled” thymocytes in H2-O WT (Top) or H2-O KO (Bottom). (Right) Summary plots of >5 repeat experiments. **(D)**. Frequency of signaled CCR7+ medulla CD4 T cells in H2-O WT (white) and H2-O KO (red) mice.

Supplementary Figure 4 Top: breakdown of H2-O KO (right) and H2-O WT (Left) Seurat clustering Bottom: Seurat clustering for each individual biological replicate.

Supplementary Figure 5 Key genes in Clusters 0-4. (Top) Top genes (Avg(log_2_)FC >1, p-value<0.05) for clusters 0-4 in concatenated data. (Bottom) Key gene expression broken into H2-O WT (left) or H2-O KO (right).

Supplementary Figure 6 Rorc expression is found within H2-O KO Tregs. Red outline indicates that minor RORy expression was found in H2-O KO cluster 2.
