# Supplementary figures and images for: H2-O deficiency promotes regulatory T cell differentiation and CD4 T cell hyperactivity
Source: Front Immunol. 2024 Jan 5;14:1304798. doi: 10.3389/fimmu.2023.1304798 (PMC10796743; doi:10.3389/fimmu.2023.1304798)

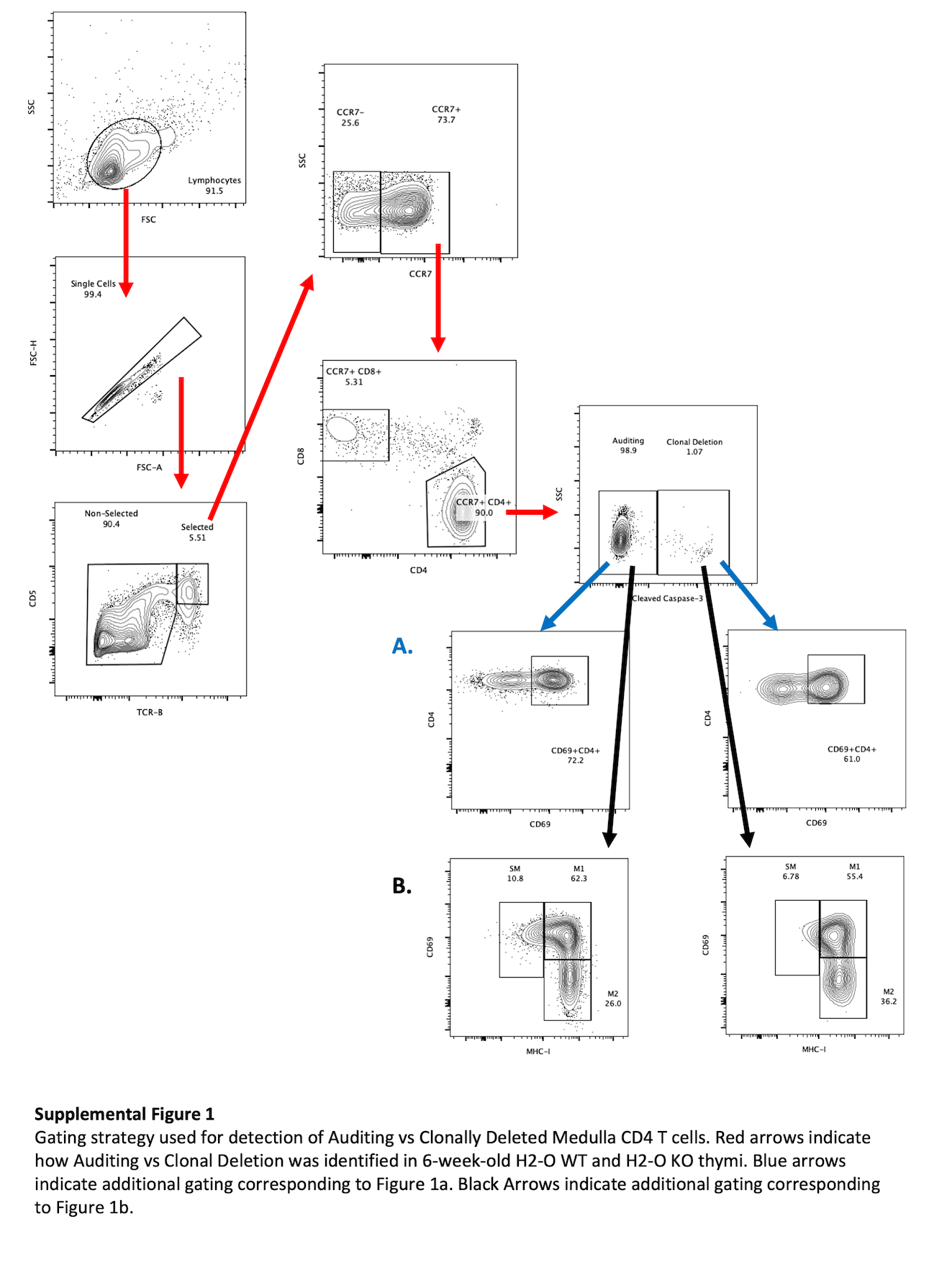

Supplement: Supplementary Figure 1 — Gating strategy used for detection of Auditing vs Clonally Deleted Medulla CD4 T cells Red arrows indicate how Auditing vs Clonal Deletion was identified in 6-week-old H2-O WT and H2-O KO thymi. Blue arrows indicate additional gating corresponding to . Black Arrows indicate additional gating corresponding to ( Figure 1B ). [file Image_1.tiff]

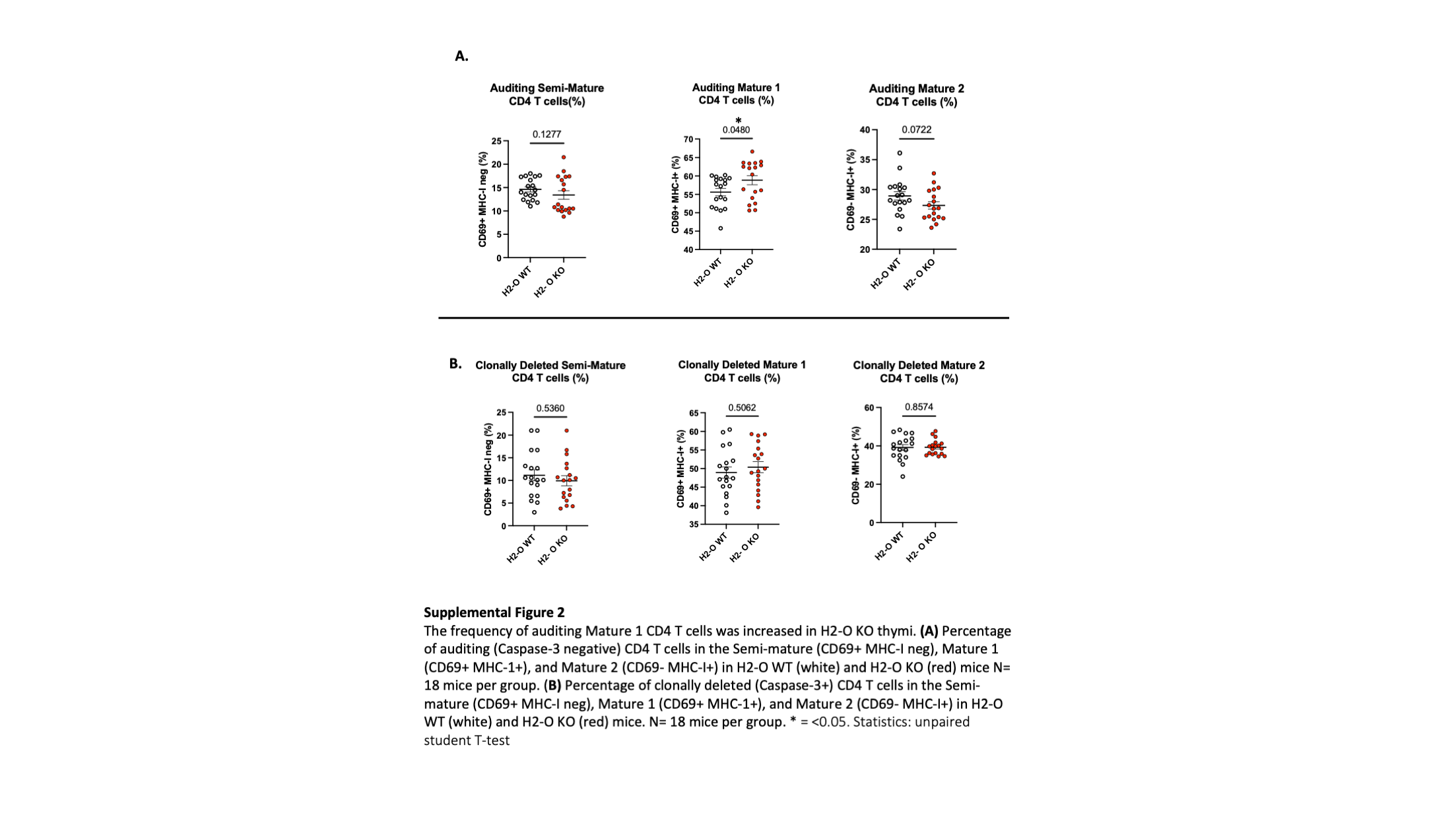

Supplement: Supplementary Figure 2 — The frequency of auditing Mature 1 CD4 T cells was increased in H2-O KO thyme (A). Percentage of auditing (Caspase-3 negative) CD4 T cells in the Semi-mature (CD69+ MHC-I neg), Mature 1 (CD69+ MHC-1+), and Mature 2 (CD69- MHC-I+) in H2-O WT (white) and H2-O KO (red) mice N= 18 mice per group (B). Percentage of clonally deleted (Caspase-3+) CD4 T cells in the Semi-mature (CD69+ MHC-I neg), Mature 1 (CD69+ MHC-1+), and Mature 2 (CD69- MHC-I+) in H2-O WT (white) and H2-O KO (red) mice. N= 18 mice per group Statistical Testing: unpaired student T-test. [file Image_2.tiff]

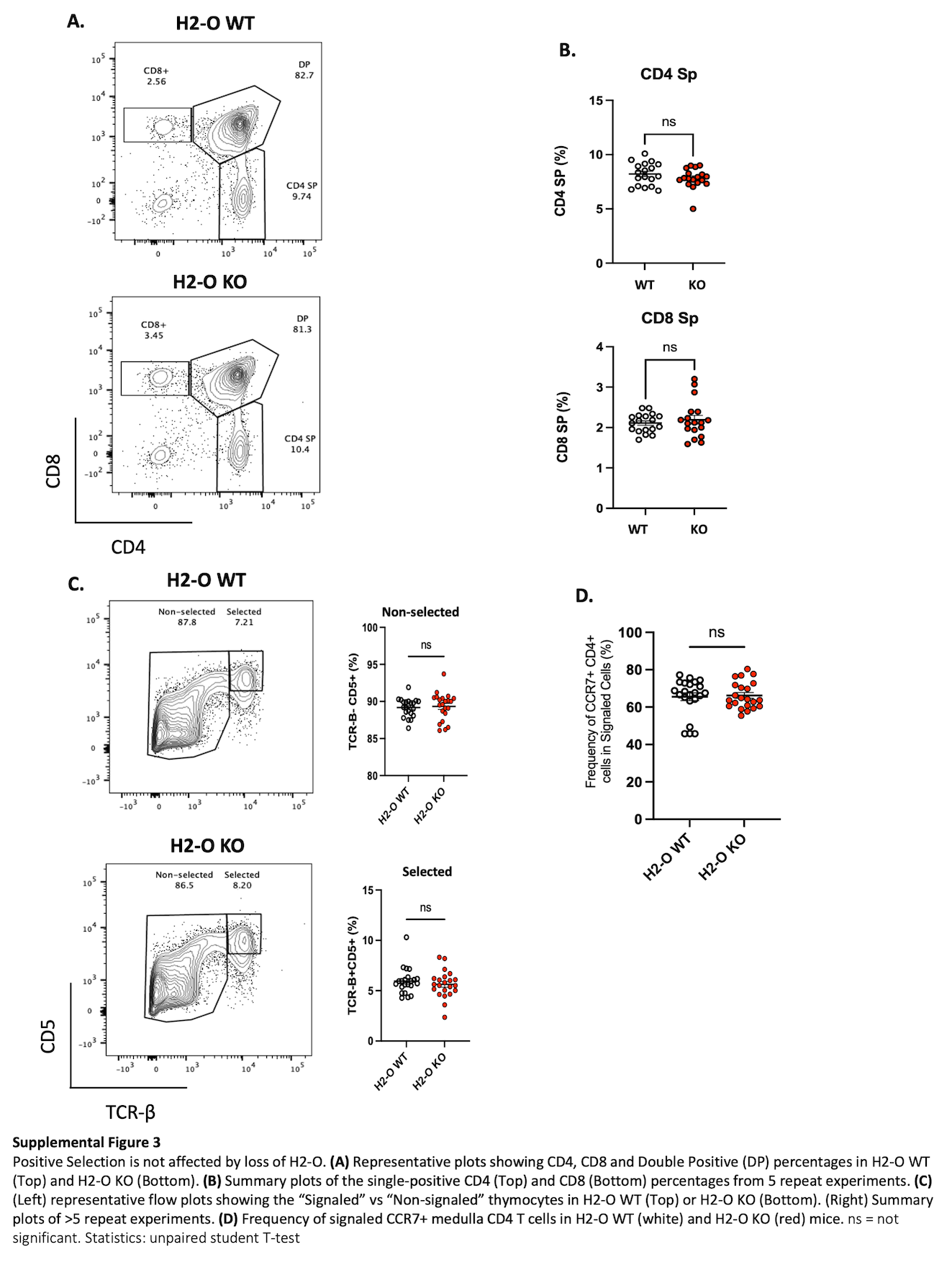

Supplement: Supplementary Figure 3 — Positive Selection is not affected by loss of H2-O (A). Representative plots showing CD4, CD8 and Double Positive (DP) percentages in H2-O WT (Top) and H2-O KO (Bottom) (B). Summary plots of the single-positive CD4 (Top) and CD8 (Bottom) percentages from 5 repeat experiments. (C). (Left) representative flow plots showing the “Signaled” vs “Non-signaled” thymocytes in H2-O WT (Top) or H2-O KO (Bottom). (Right) Summary plots of >5 repeat experiments. (D). Frequency of signaled CCR7+ medulla CD4 T cells in H2-O WT (white) and H2-O KO (red) mice. [file Image_3.tiff]

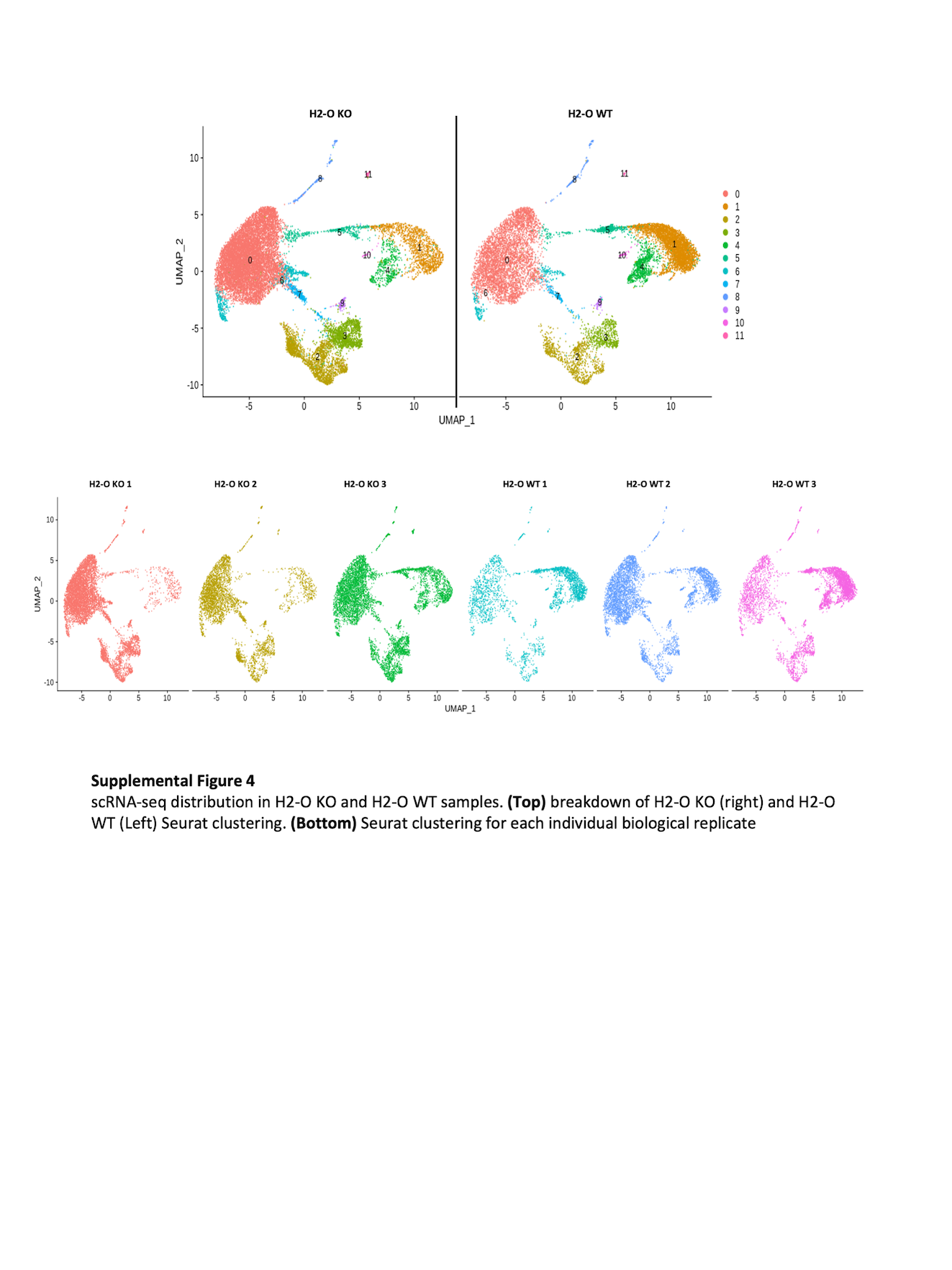

Supplement: Supplementary Figure 4 — Top: breakdown of H2-O KO (right) and H2-O WT (Left) Seurat clustering Bottom: Seurat clustering for each individual biological replicate. [file Image_4.tiff]

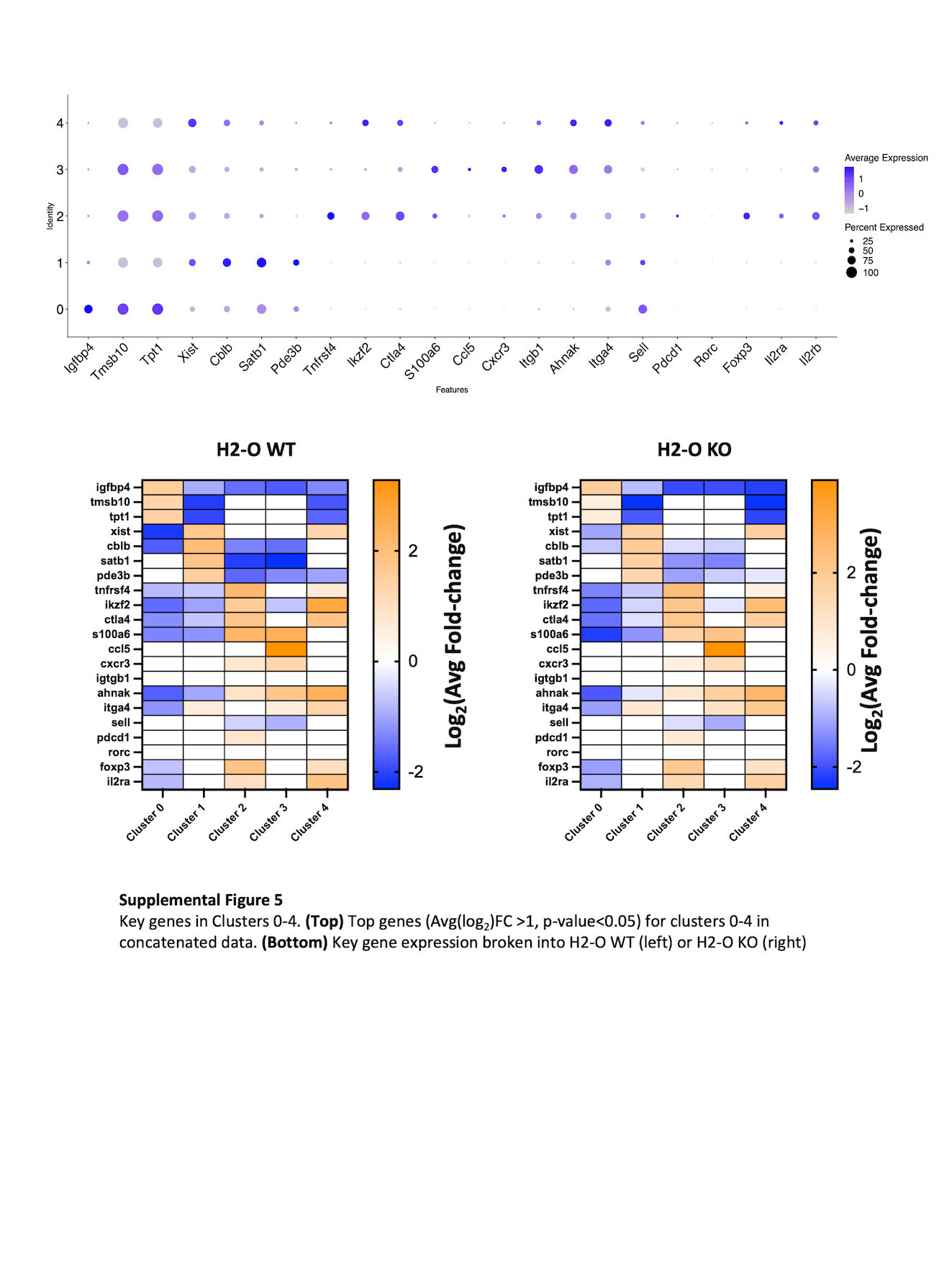

Supplement: Supplementary Figure 5 — Key genes in Clusters 0-4. (Top) Top genes (Avg(log2)FC >1, p-value<0.05) for clusters 0-4 in concatenated data. (Bottom) Key gene expression broken into H2-O WT (left) or H2-O KO (right). [file Image_5.tiff]

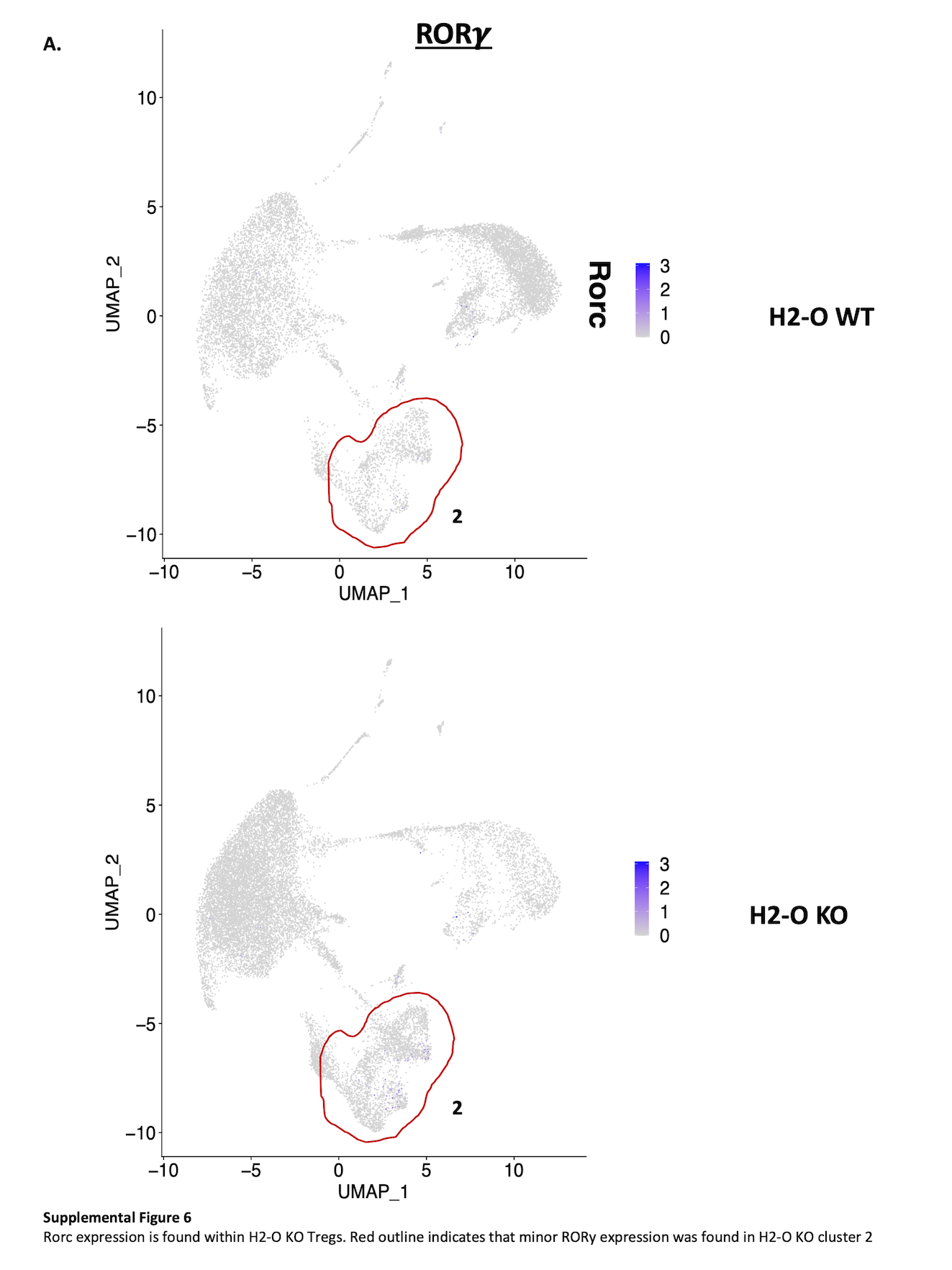

Supplement: Supplementary Figure 6 — Rorc expression is found within H2-O KO Tregs. Red outline indicates that minor RORy expression was found in H2-O KO cluster 2. [file Image_6.tiff]
